# Development of innovative computer software to facilitate the setup and computation of water quality index

**DOI:** 10.1186/2052-336X-11-1

**Published:** 2013-04-26

**Authors:** Ramin Nabizadeh, Maryam Valadi Amin, Mahmood Alimohammadi, Kazem Naddafi, Amir Hossein Mahvi, Samira Yousefzadeh

**Affiliations:** 1 Department of Environmental Health Engineering, School of Public Health, Tehran University of Medical Sciences, P.O. Box 14155-6446, Tehran, Iran; 2 Center for Water Quality Research, Institute of Environmental Research, Tehran University of Medical Sciences, Tehran, Iran

**Keywords:** Drinking water, Water quality standards, Water quality index, Water quality software, Monitoring programme

## Abstract

**Background:**

Developing a water quality index which is used to convert the water quality dataset into a single number is the most important task of most water quality monitoring programmes. As the water quality index setup is based on different local obstacles, it is not feasible to introduce a definite water quality index to reveal the water quality level.

**Findings:**

In this study, an innovative software application, the Iranian Water Quality Index Software (IWQIS), is presented in order to facilitate calculation of a water quality index based on dynamic weight factors, which will help users to compute the water quality index in cases where some parameters are missing from the datasets.

**Conclusion:**

A dataset containing 735 water samples of drinking water quality in different parts of the country was used to show the performance of this software using different criteria parameters. The software proved to be an efficient tool to facilitate the setup of water quality indices based on flexible use of variables and water quality databases.

## Introduction

The Water Quality Index (WQI) is introduced as a mathematical instrument to convert the water quality dataset into a single number which represents the water quality level while eliminating subjective assessments of water quality and biases of individual water quality experts [1]. Application of water quality indices allows the assessment of changes in water quality over time and space and also the evaluation of the efficacy of domestic policies and international strategies designed to protect aquatic resources [2]. Water quality indices are also used for the classification of water [3].

Ramakrishnaiah et al. [4] presented a groundwater WQI which was based on 12 parameters: pH, Total Hardness(TH), Ca^++^, Mg^++^, HCO_3_^−^, Cl^−^, NO_3_^−^, SO_4_^−^, Total Dissolved Dolids (TDS), Fe^++^, Mn^++^, and F^−^. According to their presented method, the values of these 12 parameters should be monitored to calculate the WQI. Relative weight factors of the mentioned twelve parameters should also be calculated, and there is no way to calculate the WQI when parameters included in the computation of the index are missing from the datasets. In many countries, water monitoring programmes are decentralized and different water monitoring sectors include their choices of parameters in routine periodic sampling and analysis. Therefore, the use of water quality indices which are based on fixed parameters overlooks large data records during the process of computing the WQI, especially when the index is not defined according to available data in the database. In many areas, especially those with extensive use of agro-chemicals, it is necessary to consider pesticides as health-risk-based parameters. Furthermore, in industrialized areas with high levels of potentially harmful anthropogenic pollutants, the role of organic solvents such as carbon tetrachloride, trichloroethylene, and perchloroethylene as potential criteria pollutants should not be overlooked; otherwise, some particular water sources may receive good scores and yet have water quality impaired by parameters not included in the index.

According to the above mentioned cases, it is not practical to set up a WQI with definite criteria pollutants which could be effectively used in all cases. Therefore, software is needed to enable water quality experts to set up their own water quality indices. Furthermore, facilities should be presented for the efficient use of parameters in water quality datasets which contain missing values. In this study, a software named as the Iranian Water Quality Index Software (IWQIS) was developed to address these issues.

## Materials and methods

### Water quality index background

Two indices were calculated in 1988; the degree of contamination for health-risk -based parameters (F^−^,NO3^−^, UO2^2−^, As, B, Ba, Cd, Cr, Ni, Pb, Rn, and Se), and the degree of contamination for technical-aesthetic parameters (pH, KMnO4 consumption, SO_4_^−^, Cl^−^, Ag, Al, Cu, Fe, Mn, Na, and Zn) [5]. In another study, nine variables were considered: nitrate, phosphate, chloride, TDS, biological oxygen demand, cadmium, chromium, nickel, and lead [6].

Stigter et al. [7] created a groundwater quality index (GWQI) with a method based on multivariate analysis for monitoring the influence of agriculture using parameters of groundwater chemistry and potability and tested its applicability in the south of Portugal. They included nitrate, sulphate, chloride, and calcium in their presented index. A groundwater quality index (GWQI) was also developed to assess water quality affected by a landfill site based on seven variables [8]. In this study, creation of the index was based on Principal Component Analysis (PCA) and benchmarking analysis. They showed that seven variables, electric conductivity, TDS, salinity, nitrate, chemical oxygen demand, and iron, could be used as indicators. Simoes et al. [9] proposed a Water Quality Index for management purposes in the Medio Paranapanema Watershed in Sao Paulo State, Brazil, as a pollution indicator for aquaculture activity based on three parameters: turbidity, total phosphorus, and dissolved oxygen. They showed that the water quality degradation in the studied area due to aquaculture activity could be described with this simple index.

The groundwater quality in Sunamganj, Bangladesh, was studied based on different indices for irrigation and drinking uses. Parameters such as absorption ratio, soluble sodium percentage, residual sodium carbonate, electrical conductance, magnesium adsorption ratio, Kelly’s ratio, total hardness, permeability index, and residual sodium bi-carbonate were included to investigate the ionic toxicity [10].

Terrado et al. [11] selected the WQI of the Canadian Council of Ministers of the Environment (CCME WQI) as the most suitable index. It gives a number between 0 (worst quality) and 100 (best quality). They also performed a sensitivity analysis for the CCME WQI to select the best procedure for optimizing the WQI according to input data. Sharma and Patel [12] collected various seasonal groundwater samples for some consecutive years and the respective physiochemical analysis was carried out for five groundwater quality parameters (pH, TDS, chlorides, hardness, and electrical conductivity) which are essentially responsible for groundwater quality degradation in the studied area. They indicated that the groundwater of the study area needs to achieve a considerable degree of quality improvement by the most feasible approach such as artificial groundwater recharging. Yidana et al. [13] developed a groundwater classification scheme using a robust WQI modified for the case of the Keta basin and classified groundwater in their study area into ‘good’, ‘fair’, and ‘marginal’ water types using ordinary kriging developed from a well fitted linear semivariogram function. Recently, a global, country-level Water Quality Index (WATQI) was developed as a research and policy-making tool for the measurement and management of freshwater quality based on data from the UNEP GEMS/Water programme and the European Environment Agency (EEA) [14].

Omo-Irabor et al. [15] subjected the chemical data set to PCA/FA, and Hierarchic Cluster Analysis (HCA). The aim of this study was to determine the nature and spatial distribution of chemical pollutants in surface and groundwater resources in the western Niger Delta region. Yidana et al. [16] used the multivariate method to analyse surface water hydrochemical data from different locations along the Ankobra Basin, Ghana. They aimed to extract principal factors related to different sources of variation in the hydrochemistry, and therefore they combined PCA and CA to classify water samples into specific groups on the basis of hydrochemical characteristics. Banoeng-Yakubo et al. [17] calculated a WQI for samples using concentrations of Na^+^, Ca^++^, Mg^++^, Cl^−^, NO_3_^−^, F^−^, and EC at various sample locations. R-mode HCA and factor analysis (using varimax rotation and the Kaiser Criterion) were used to find the significant sources of variation in the hydrochemistry. They classified the WQI values into five categories as follows (<50: excellent water; 50–100: good water; 100–200: poor water; 200–300 very poor water; >300: water unsuitable for drinking). Saeedi et al. [18] used a WQI to analyse the nature and rate of land use change and its associated impact on groundwater quality. In this study, a methodology based on multivariate analysis was developed to create a GWQI that aimed to identify the places with the best quality water for drinking within the Qazvin province in western central Iran. Al-Shami et al. [19] studied the abundance and diversity of benthic macroinvertebrates as well as physico-chemical parameters in five rivers of the Juru River Basin in northern Peninsula Malaysia. The physico-chemical parameters and calculated WQI were significantly different among the investigated rivers (ANOVA, p < 0.05). They concluded that the multivariate analysis (CCA) was highly satisfactory, explaining 43.32% of the variance for the assemblages of macroinvertebrates as influenced by 19 physical and chemical variables.

Bu et al. [20] studied the sampled water quality at 12 sampling sites in the Jinshui River of the South Qinling Mountains in China. It was confirmed that 25 studied water quality variables had significant temporal differences (p < 0.01) and spatial variability (p < 0.01). Based on the similarity of water quality variables and application of cluster analysis, the 12 sampling sites were classified into three pollution level groups (no pollution, moderate pollution, and high pollution). Razmkhah et al. [21] applied PCA and HCA methods to determine the water quality of Jajrood River (Iran) and to assess and discriminate the relative magnitude of anthropogenic and natural influences on the quality of river water. T, EC, pH, TDS, NH_4_^+^, NO_3_^−^, NO_2_^−^, Turbidity, Total Hardness, Ca^++^, Mg^++^, Na^+^, K^+^, Cl^−^, SO_4_^−^, and SiO_2_ were selected as the physico-chemical variables and total coliform and faecal coliform as the biochemical variables to be analysed in the water samples from 18 sampling stations.

In another study, parameters such as dissolved oxygen (DO), biochemical oxygen demand (BOD), pH, temperature, TDS, turbidity, faecal coliform, heterotrophic plate count, hardness, alkalinity, arsenic, lead, mercury, nickel, cadmium, chromium, total phosphorous, H_2_S, nitrate, and fluoride were selected to develop the quality of drinking water supplied to dairy cattle based on fuzzy logic using trapezoidal membership functions [22]. In our recent study, we selected twenty parameters which were included based on their critical importance for the overall water quality and their potential impact on human health to assess the performance of the proposed index under actual conditions. The comparison of the outputs of the fuzzy-based proposed index with those of the NSF WQI and Canadian Water Quality Index (CWQI) showed similar results and were sensitive to changes in the level of water quality parameters [23].

### Water quality index setup

The structure of variables, weights, mathematical relationships, and specific features of the GWQI presented in this study are described in this section. For different water quality indices, various variables may be selected according to the importance of the parameters and availability of data. In this study, we developed software which enables users to choose different parameters according to the desired criteria pollutants. In the software, the user can select up to 40 variables which are supposed to be responsible for water contamination based on the importance of the variables, the availability of data, and experts’ professional judgements. The most frequently used variables in other studies which are used in water monitoring programmes and in our national monitoring water activities are set as default parameters.

In this study, we tested the performance of IWQIS on a database with 735 water samples from different drinking water resources in the country. The selected parameters, weights, and limit values which were used to set up the WQI for the mentioned dataset are presented in Table 1. Ramakrishnaiah et al. [4] selected total hardness, calcium, and magnesium in their index. Although the total hardness data were available during the setting of the WQI, we did not include it in the criteria parameters, since the total hardness could be calculated by calcium and magnesium and including the calcium, magnesium, and total hardness at the same time would cause bias in the computation of the WQI. It should be noted that interpretation of the calculated WQI was performed according to the classification presented in Table 2, which was presented by Sharma and Patel [12].

**Table 1:** Criteria parameters, weight factors, and limit values considered for setting up the water quality index

**Criteria parameters**	**Weights**	**Limit values(mg/L)**
pH	4	6.5–8.5
Calcium	2	300
Magnesium	2	30
Chloride	3	250
TDS	4	1000
Fluoride	4	1.5
Manganese	4	0.1
Nitrate	5	50
Iron	4	0.3
Sulfate	4	250
Ammonium	3	1.5
Sodium	3	200
Turbidity	4	5

**Table 2:** Water quality classification based on WQI values

**Water quality index values**	**Interpretation**
<50	Excellent water quality
50–100	Good water quality
100–200	Poor water quality
200–300	Very poor water quality
>300	Unsuitable for drinking

The main concept and incentive for developing the IWQIS was to facilitate the computation of WQI with more flexibility and to make the calculation of the WQI feasible in cases where some data related to selected criteria pollutants are missing from the database. It is very common to find missing values in some records of water quality databases. As mentioned, all the previous water quality indices were based on the use of fixed parameters and their definite weights. The practical shortcoming of these indices appears when one or more parameters are not available in a record set. In these cases the other data could not be used for calculation of the index, since the weights are fixed and cannot be changed. In the method presented in this study, the weights are dynamic and in cases where users face a lack of data in records of water samples, the new relative weights are recalculated according to the available data.

**Figure 1:**
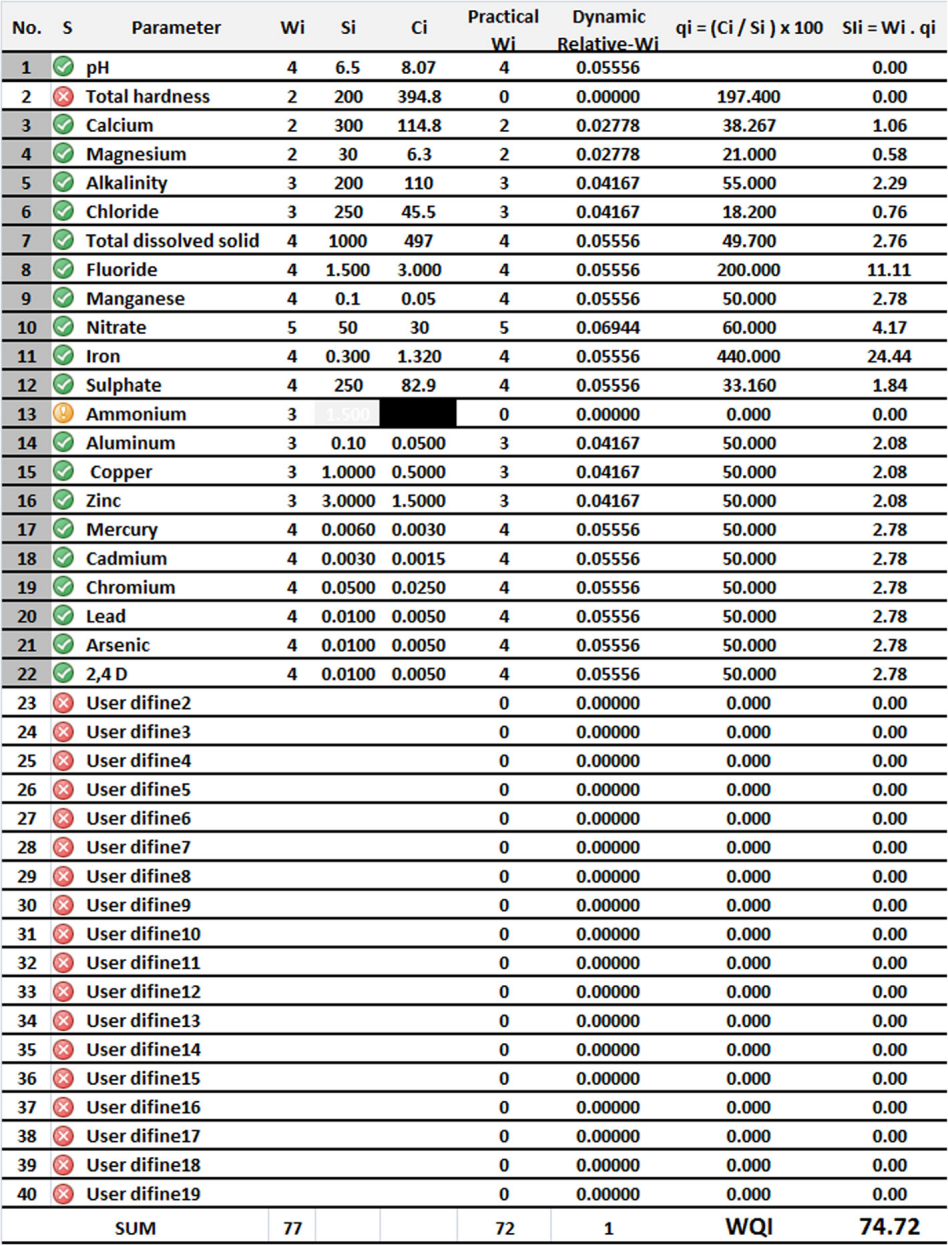
The concept of dynamic relative risks for a single sample in excel datasheet.

The concept of dynamic relative risks is illustrated in Figure 1 for a single record in an Excel data sheet (which can be downloaded from http://tums.ac.ir/ajaxplorer/data/public/2941eae50882f1adcb47436ef78c0e16.php?lang=en) to familiarize readers with the presented index and the idea of using dynamic weights for computation of the WQI.

As shown in this example, the user has selected 21 items as criteria parameters. Total Hardness is excluded by typing −1 in the column S (indicated by ×) and 20 parameters and 2,4 D (as user define 1) are included by typing 1 (indicated by √) in the column S. Although ammonium was initially selected as a criterion criteria parameter, it was excluded from the process in this single record because there were no data for this parameter. To exclude a parameter for which there is no available data, the user can simply type a zero in column S (indicated by !). Dynamic relative weights would be recalculated according to the selected parameters to compute the WQI. In this way, the data in a dataset which may have some missing values for some parameters could be effectively used. For pH, the quality value is considered zero when the pH is between 6.5 and 8.5. For pH values less than 6.5, the quality value is computed according to the following formula:

(1) $$\mathrm{qvalue}\_\mathrm{pH}=\left(6.5/\mathrm{pH}\;*\;100\;\right)\;*\;\mathrm{Dynamic}\mathrm{Weight}\mathrm{of}\mathrm{pH}$$

and for pH values greater than 8.5, it is calculated through the following relationship:

(2) $$\mathrm{qvalue}\_\mathrm{pH}=\left(\mathrm{pH}/8.5\;*\;100\right)\;*\;\mathrm{Dynamic}\mathrm{Weight}\mathrm{of}\mathrm{pH}$$

For the other parameters, quality values are calculated according to the following formula.

(3) $$\begin{array}{ll} \;\mathrm{Quality}\mathrm{value}= & \left(\mathrm{Conc}.\;\mathrm{of}\mathrm{parameter}/\mathrm{Limit}\;\mathrm{Value}\;*\;100\right)\\ & *\;\mathrm{Dynamic}\mathrm{Weight}\mathrm{of}\mathrm{parameter} \end{array}$$

As previously mentioned, quality scores are determined dynamically for parameters which have available data in the water quality dataset. The water quality index, a dimensionless number, is determined as the sum of all quality values for those constituents chosen by the user as criteria parameters.

## Software specification

In this study, user-friendly software has been developed according to the concept of dynamic weights allocation to make the computation of the WQI simple. This package is called the Iranian Water Quality Index Software (IWQIS) and can be effectively used to process water quality data according to the user’s choice of parameters, weights, and limit values. The authors provide access to the mentioned software (via: http://tums.ac.ir/ajaxplorer/data/public/ec21f02fcf2f681a89a2f7500c83d1e6.php?lang=en) in order to simplify the water quality assessment monitoring activities.

**Figure 2:**
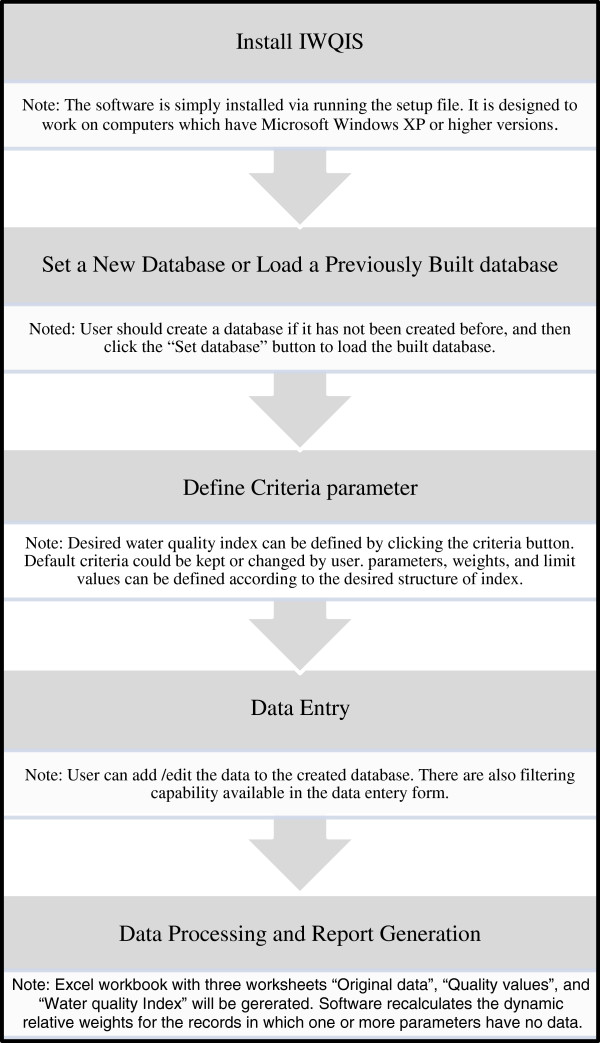
Diagram to illustrate how the software works.

In this section, the software requirements, capabilities, and application are described. Figure 2 illustrates how the software works. It is simply installed by running the setup file which is designed to work on computers with Microsoft Windows XP or more recent versions. Users should also have Microsoft Excel installed on their computers, since the reports are designed to be transferred in Excel workbooks. Excel reports enable the user to perform additional analysis on the output files.

**Figure 3:**
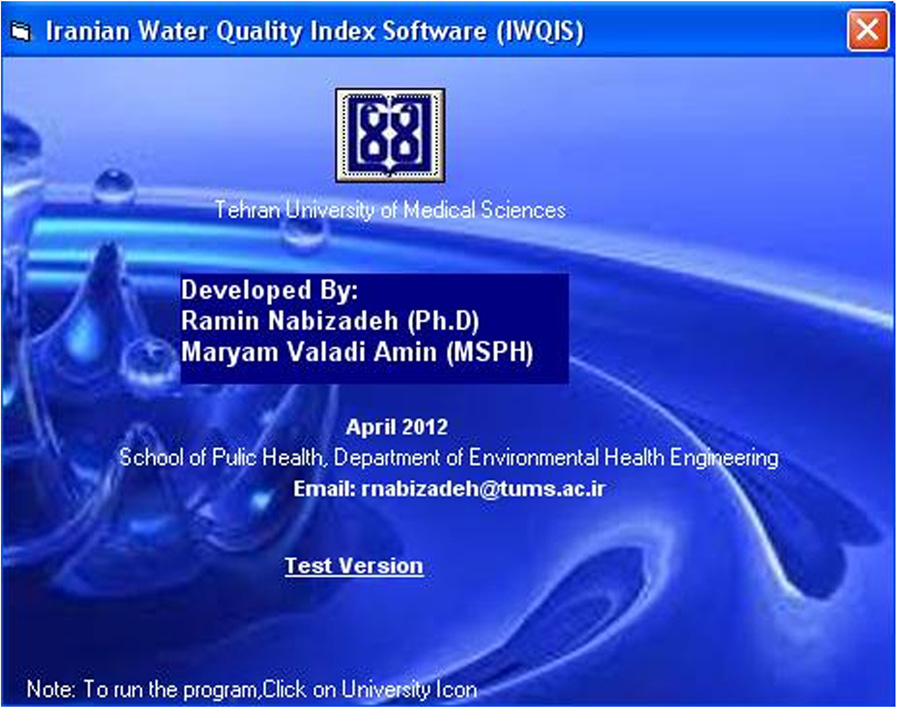
The main window of IWQIS.

As shown in Figure 3, when the user clicks on the icon, the program starts and the form appears. Using this form the user is able to set a new database or load a previously built database.

**Figure 4:**
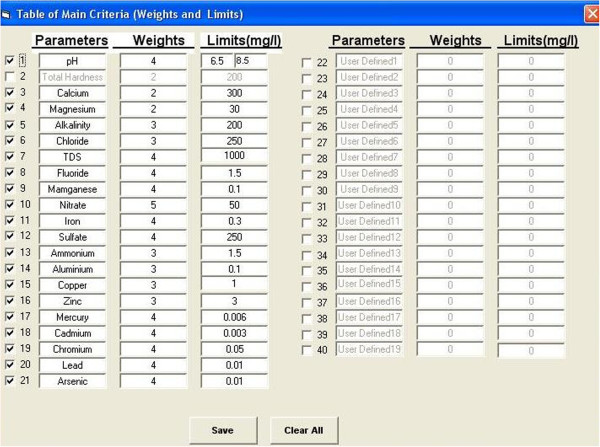
Facilities of IWQIS provided for the user to define the criteria parameter, weights, and limit values.

It should be noted that the user should create a database if it has not been created before, and then click the “Set database” button to load the built database. The criteria parameters of the desired WQI can be defined by clicking the criteria button. Figure 4 shows the facilities which are provided for the user to define the criteria parameters of the index. In this form, parameters, weights, and limit values can be defined according to the desired structure of the index. In this software, 21 parameters are set as default which can be selected by user. There are also 19 user-defined parameters which can be set as criteria parameters based on the availability of data and experts’ professional judgements. After defining the parameters, weights, and limit values, the user can add the data to the created database and generate an output report.

The report of IWQIS is generated as an Excel workbook with three worksheets, “Original Data”, “Quality Values”, and “Water Quality Index”. The first sheet, Original Data, includes the data which were previously entered in the database. The second sheet presents the calculated quality values for each parameter and the third sheet includes WQI and the related interpretations.

### Spatial variability and principal component analysis

Recently, multivariate statistical methods have been used to characterize and evaluate surface and groundwater. Chemical, biological, and physical data were monitored at 12 locations along the Passaic River, New Jersey and analysed in a study performed by Bengraine and Marhaba [24]. PCA was used to extract the factors related to the hydrochemical variability and to demonstrate the spatial and temporal changes in water quality. Singh et al. [25] used cluster analysis (CA), factor analysis (FA), PCA, and discriminant analysis (DA) of the dataset on water quality of the Gomti River (India). They concluded that 10 parameters (river discharge, pH, BOD, Cl, F, PO_4_, NH_4_–N, NO_3_^–^N, TKN, and Zn) contributed to 97% correct assignations in the spatial analysis of three different regions in the basin. Zhou et al. [26] showed that multivariate statistical methods are useful for interpreting complex data sets in the analysis of temporal and spatial variations in water quality and could be used for the optimization of a regional water quality monitoring network.

In this study, the spatial variability in the dataset with 735 drinking water samples in the country was illustrated using box plots. After filtering records with missing values, PCA was performed to find the meaningful components. The retained components were used to perform a linear model. Finally, the fitness of predictions of the principal component model generated and the WQI computed by IWQIS was determined. It should be noted that PCA was performed using R software [27].

## Results and discussion

Stambuk-Giljanovic [1] believes that lack of consent for the selection of quality evaluation parameters is the greatest obstacle to a broader index application in the world. Rickwood and Carr [2] published a list of all possible parameters, their associated WHO guidelines, and whether they were measured in 20%, 35%, and 50% of countries in all regions: Europe, Asia, Africa, Americas, and Oceania. The appropriate selection of criteria variables from the list for setting the quality index is still the most important task. In this study, the selection of variables was essentially based on the availability of data on the national scale. We tried to choose those parameters which are commonly measured in water monitoring programmes. In this stage, the objective of our study was to show a general picture of drinking water quality using widely selected water samples from around the country.

**Figure 5:**
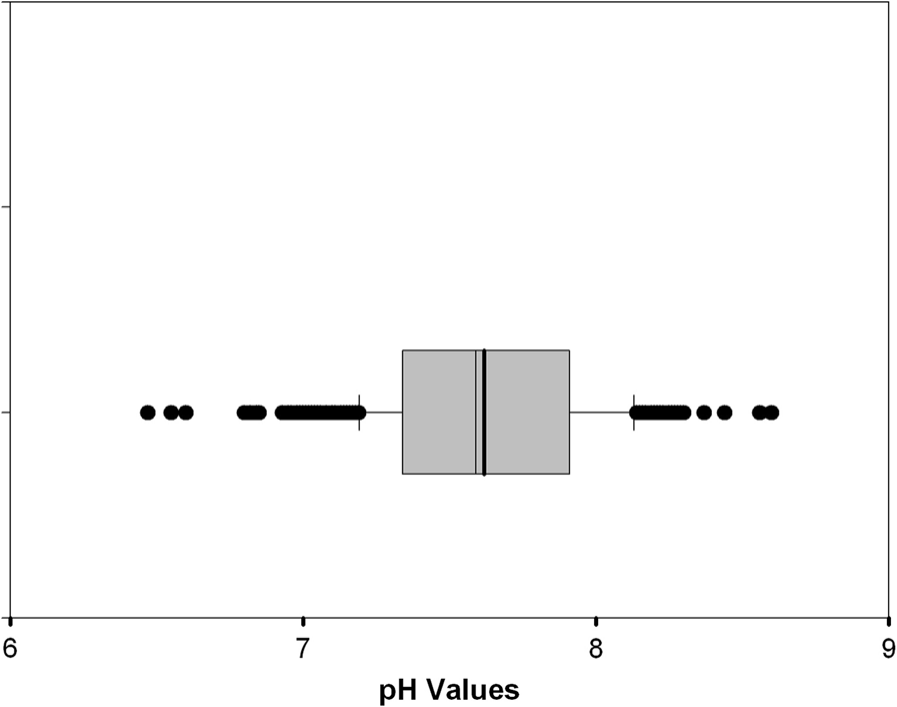
Spatial variability for pH.

**Figure 6:**
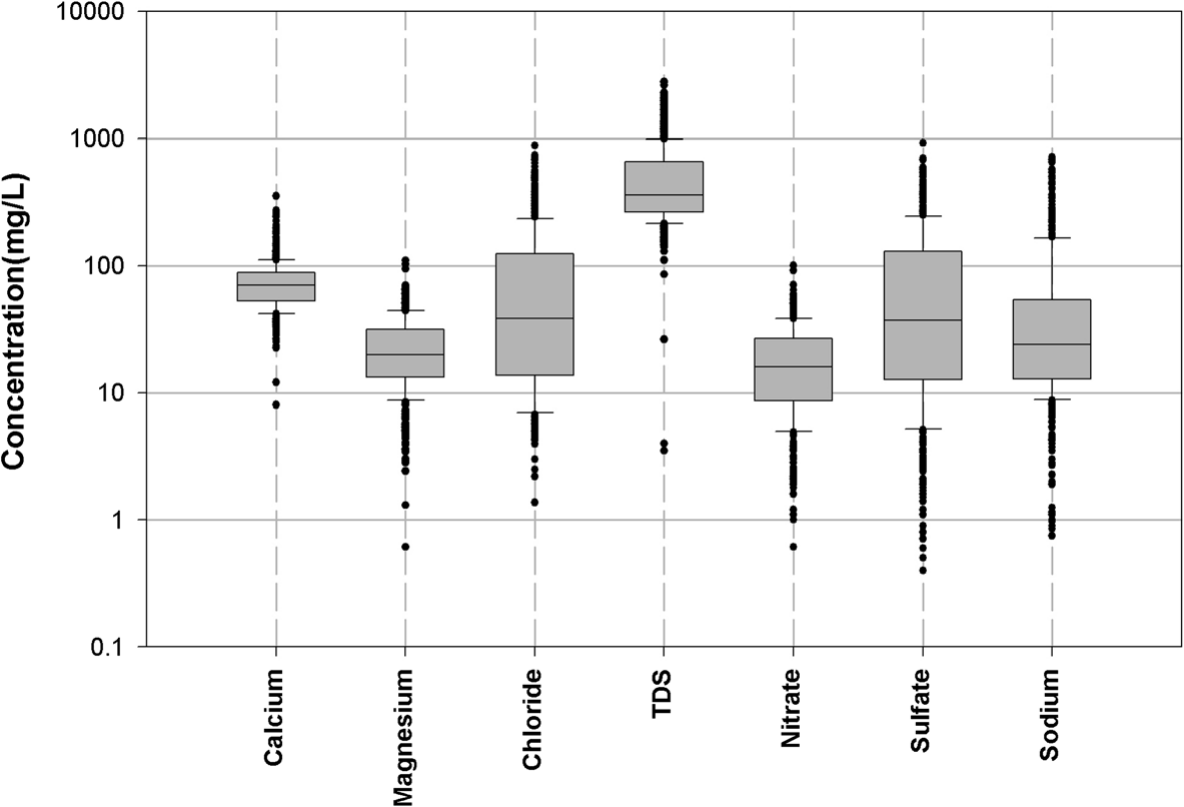
Spatial variability for calcium, magnesium, chloride, TDS, nitrate, sulfate, and sodium.

**Figure 7:**
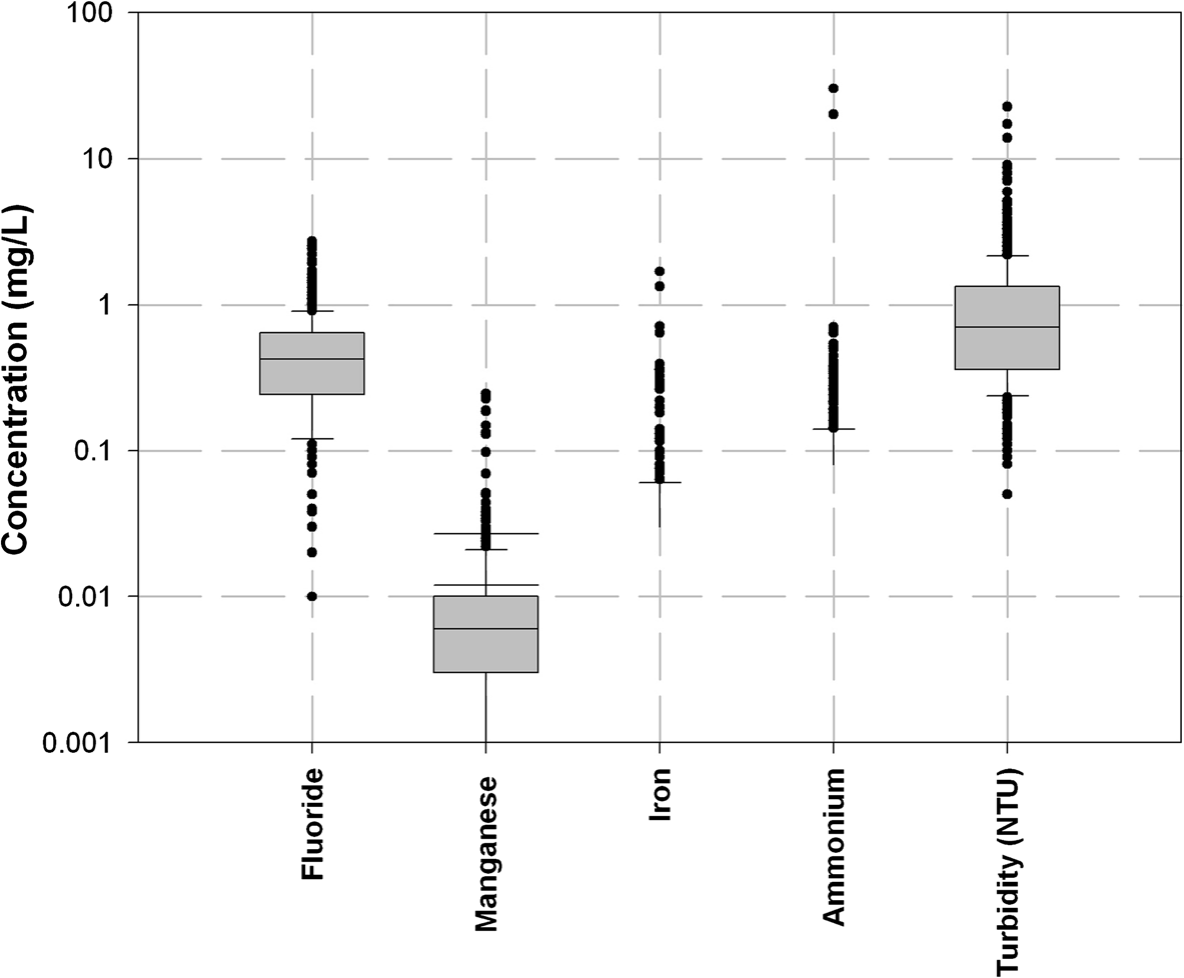
Spatial variability for fluoride, manganese, Ammonium, and turbidity.

Figures 5, 6 and 7 show the spatial variability of drinking water quality parameters in the database.

Multivariate analysis carried out by means of PCA resulted in four components, which accounted for 74% of the spatial changes. Table 3 presents standardized loadings based upon the correlation matrix in the applied PCA. As shown in Table 3, the components having SS loadings or eigenvalues greater that one were retained. The first component accounts for about 44% of the variance, the second component for about 13%, and the third and fourth for about 10% and 8%, respectively.

**Table 3:** Standardized loadings (pattern matrix) based upon correlation matrix

**p**	**PC1**	**PC2**	**PC3**	**PC4**
SS loadings	6.09	1.76	1.34	1.19
Proportion Var	0.44	0.13	0.1	0.08
Cumulative Var	0.44	0.56	0.66	0.74
Proportion explained	0.59	0.17	0.13	0.11
Cumulative proportion	0.59	0.76	0.89	1

Table 4 presents the loading of each variable under each of the four components. The first principal component represents the most important process or mixed process controlling the hydrochemistry, which has the highest eigenvalue and accounts for the highest variance in the component matrix.

**Table 4:** Component matrix

**Parameters**	**PC1**	**PC2**	**PC3**	**PC4**	**h2**	**u2**
pH	0.38	−0.58	−0.15	0.16	0.53	0.469
Calcium	0.29	***0.71***	0.06	−0.12	0.61	0.389
Magnesium	***0.76***	0.17	0.11	−0.11	0.64	0.364
Chloride	***0.96***	−0.07	−0.03	0.01	0.93	0.075
TDS	***0.98***	−0.06	−0.02	0.01	0.96	0.043
Fluoride	***0.90***	−0.07	−0.04	0.01	0.81	0.19
Manganese	0.12	***0.73***	−0.3	0.12	0.66	0.342
Nitrate	−0.17	0.42	***0.69***	0.02	0.68	0.323
Iron	−0.24	0.17	−0.35	0.01	0.21	0.79
Sulfate	***0.89***	0.31	−0.06	0.01	0.89	0.107
Ammonium	0.04	−0.08	0	***0.98***	0.96	0.035
Sodium	***0.95***	−0.06	−0.09	0.05	0.92	0.083
Turbidity	−0.06	−0.1	***0.76***	0	0.6	0.404

In this study, the first component, which accounts for about 44% of the variance, has high positive loadings for magnesium, chloride, TDS, fluoride, and sulfate, and could be due to the dominant share of groundwater resources in supplying drinking waters. The second principal component accounts for about 13% of the hydrochemistry and has a high positive loading for calcium. This factor could be related to higher alkalinity of groundwater due to bicarbonate ions. The third principal component accounts for about 10% of the variance in the hydro-chemical data and has high positive loadings for NO_3_^−^ and turbidity. This could be attributed to the impact of domestic waste and agricultural activities. The fourth principal component represents about 8% of the variance in the hydrochemistry of drinking water in the country and has high positive loadings for ammonium, which is an indication of agricultural practice with excessive use of fertilizers.

Using the factor scores, a linear regression model was developed to investigate the fitness of four principal components and the WQI which was computed by IWQIS. The outputs of the linear regression model for the four retained principal components are presented in Table 5. The low p-value (< 0.05) indicates the significance of the model. The high value of multiple R-squared (0.9883) shows the strong correlation between WQI values and predictions from the principal components model.

**Table 5:** Outputs for linear model of 4 retained principal components

**Coefficients**	**Estimate**	**Std. error**	**t**	**value Pr(>|t|)**
(Intercept)	29.5616	0.1157	255.40	<2e-16 ***
scores$PC1	18.6690	0.1159	161.08	<2e-16 ***
scores$PC2	2.1963	0.1159	18.95	<2e-16 ***
scores$PC3	2.2290	0.1159	19.23	<2e-16 ***
scores$PC4	8.3036	0.1159	71.64	<2e-16 ***

Signif. codes: 0 ‘***’ 0.001 ‘**’ 0.01 ‘*’ 0.05 ‘.’ 0.1 ‘’ 1.

Residual standard error: 2.259 on 376 degrees of freedom.

Multiple R-squared: 0.9883, Adjusted R-squared: 0.9882.

F-statistic: 7952 on 4 and 376 DF, p-value: < 2.2e-16.

**Figure 8:**
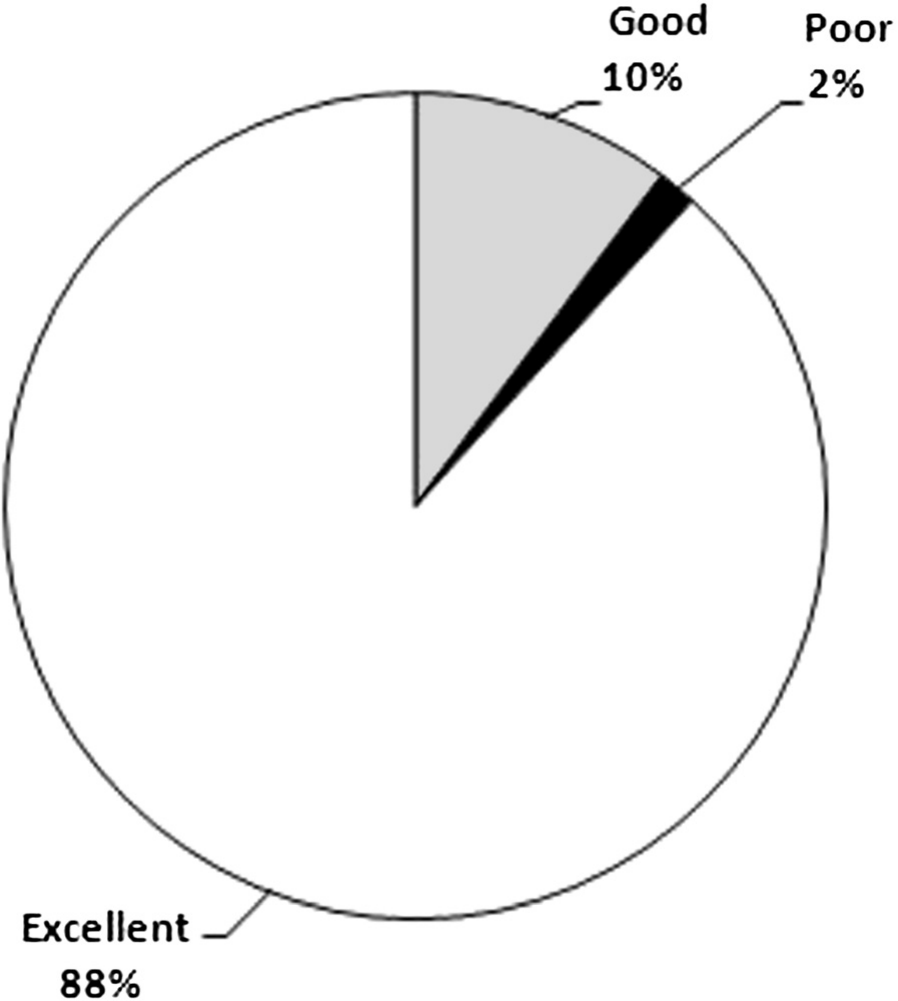
The share of each water quality classification based on WQI values.

Table 6 summarizes the descriptive statistics of the WQI which was computed for 735 drinking water samples in the study. Figure 8 illustrates the share of each water quality classification presented by Sharma and Patel [12] based on WQI values.

**Table 6:** Descriptive statistics of water quality index of drinking water samples

			
Mean	29.64	Quantile%	*WQI*
Standard Error	0.77	10	12.36
Median	23.16	25	16.685
Standard Deviation	20.75	50	23.16
Sample Variance	430.67	75	36.5
Range	155.85	90	54.416
Minimum	6.23	95	71.309
Maximum	162.08		
Sum	21787.77		
Confidence Level (95.0%)	1.50		

## Conclusion

The previous works done by researchers had revealed that water quality indices should be set according to generic water quality parameters as well as locally important variables which may not be of importance in other locations. The results of these researches showed that the WQI for the monitoring of water quality changes with time and location. Hence, the importance of the variables, availability of the data, and experts’ professional judgements should be considered as the main cornerstones of WQI development. In this study, the Iranian Water Quality Index Software (IWQIS) has been set, tested and proved to be an efficient tool to facilitate the setting up of water quality indices based on flexible use of variables and existing water quality databases. The software prepared in this work will help researchers and water quality monitoring experts to design and calculate their own water quality indices easily. The presented software can be used by other researchers and communities based on the following considerations.

• The criteria parameters, weights, and limit values should be entered into the program according to local considerations.

• If the data are previously available, IWQIS would be a helpful tool to calculate the desired WQI, especially if there are some missing values in the record set.

• In cases where samples with many parameters have been collected, techniques such as PCA are useful to reduce the number of variables.

• IWQIS can also be used to determine the sensitivity analysis of weights attributed to the parameters when the allocation of definite weight factors to some parameters is controversial.

## Competing interests

The authors confirm that there is not any competing interest in publishing the results of the study.

## Authors’ contributions

This study is a part of MSPH thesis of Mrs. Valadi Amin who prepared the literature survey and general design of software and performed data manipulation and checked the validity of final output of software. The study supervised by Dr. R Nabizadeh who is the corresponding author, presented the dynamic comutation of water quality index, and took part as the technical software generator, prepared the manuscript, and performed the principal component analysis (PCA). M. Alimohammadi, and K. Naddafi took part as consultant in selecting the required parameters and weights of each default parameters used in the water quality index. A.H. Mahvi contributed to the software optimization as a water quality expert. Testing the software was performed by S. Yousefzadeh. All authors have made extensive contribution into the review and finalization of this manuscript. All authors have read and approved the final manuscript.
